# Anatomical Considerations in the Twin Block Technique for the Treatment of Masticatory Myofascial Pain: An Anatomical Review

**DOI:** 10.3390/jcm14238299

**Published:** 2025-11-22

**Authors:** Camila Venegas-Ocampo, Veronica Iturriaga, Nicolás E. Ottone, Carlos Torres-Villar, Franco Marinelli, Ramón Gelabert, Ramón Fuentes

**Affiliations:** 1 Dental School, Research Centre for Dental Sciences (CICO), Universidad de La Frontera, Temuco 4780000, Chile; c.venegas09@ufromail.cl (C.V.-O.); nicolas.ottone@ufrontera.cl (N.E.O.); 2 Núcleo de Investigación en Ciencias de la Salud, Universidad Adventista de Chile, Chillán 3780000, Chile; dirmetodologiainvestigacion@unach.cl; 3 Department of Integral Adults Adult Dentistry, Dental School, Universidad de La Frontera, Temuco 4780000, Chile; veronica.iturriaga@ufrontera.cl; 4 Temporomandibular Disorder and Orofacial Pain Program, Sleep & Pain Research Group, Faculty of Dentistry, Universidad de La Frontera, Temuco 4780000, Chile; 5 Center of Excellence in Morphological and Surgical Studies (CEMyQ), Doctoral Program in Morphological Sciences, Universidad de La Frontera, Temuco 4780000, Chile; 6 Laboratory of Plastination and Anatomical Techniques, Universidad de La Frontera, Temuco 4780000, Chile; 7 Departamento de Ciencias Morfológicas, Facultad de Ciencias, Universidad San Sebastián, Puerto Montt 5501842, Chile; carlos.torres@uss.cl; 8 Doctoral Program in Morphological Sciences, Faculty of Medicine, Universidad de La Frontera, Temuco 4780000, Chile; 9 Facultad de Ciencias de la Salud, Universidad Autónoma de Chile, Temuco 4810101, Chile; franco.marinelli@uautonoma.cl

**Keywords:** twin block technique, masseteric nerve, deep temporal nerve, myofascial pain, temporomandibular disorders

## Abstract

Myofascial pain (MFP) is one of the most frequent temporomandibular disorders (TMDs), primarily affecting the masseter and temporalis muscles. Various treatment strategies have been developed, including trigger point injections (TrP) and nerve blocks. Among these, the twin block technique has recently emerged as a promising, minimally invasive approach for simultaneously anesthetizing the masseteric and anterior deep temporal nerves through a single extraoral injection. This review presents the anatomical considerations essential for the application of the twin block technique. The course, branching patterns, and relationships of the masseteric and deep temporal nerves with adjacent vascular structures are described based on the current anatomical literature. A comparison is also made of isolated nerve blocks and the twin block, highlighting procedural protocols, clinical advantages, and safety profiles. The anatomical proximity between the masseteric and deep temporal nerves supports the rationale for a single-puncture approach, which can effectively reduce muscle tone, inhibit nociceptive input, and silence multiple trigger points simultaneously. In addition to its therapeutic benefits, the twin block can serve as a diagnostic tool to differentiate muscular from joint or odontogenic pain. In conclusion, the twin block technique offers a precise and efficient method for managing masticatory myofascial pain, provided that detailed anatomical knowledge is applied to ensure procedural accuracy, a low incidence of adverse effects, and patient safety.

## 1. Introduction

Myofascial pain (MFP) is a non-inflammatory condition of the muscular system characterized by the presence of myofascial trigger points (MTPs) and taut bands sensitive to stimuli within the fascia of the affected muscle. The clinical presentation is consistent with musculoskeletal pain, including limited mobility, muscle weakness, a dull or pressing sensation in the affected muscle, and heterotopic pain [1,2]. The predominant explanation for the pathogenesis of MTPs is Simons’ integrated hypothesis, which posits that trigger points result from excessive acetylcholine release at the neuromuscular junction, resulting in sustained muscle fiber contraction and a concurrent local energy crisis [3]. MFP is a common condition affecting the masticatory muscles and is considered one of the most frequent temporomandibular disorders (TMDs) [4].

A wide range of treatment options is currently available, depending on the muscle(s) involved, the course of the condition, and the individual’s clinical situation. These treatments may include physical rehabilitation therapy, pharmacological therapy, dry needling, wet needling, nerve blocks, acupuncture, radiofrequency treatment, and psychotherapy, among others [5]. Needling techniques are currently among the most comprehensive and significant approaches in pain medicine [5]. They are generally categorized into two types: wet needling, which involves the injection of a specific substance into the muscle tissue, and dry needling, which refers to needle insertion without the administration of any substance [4]. The most commonly used substances in wet needling include local anesthetics, botulinum toxin, platelet-rich plasma (PRP), collagen, corticosteroids, and even normal saline solution [4,6].

Among TMDs, the most commonly affected muscles in MFP are the masseter and temporalis muscles [7]. This may be due to their tonic function and role as mandibular stabilizers, which makes them susceptible to overload. Intramuscular needling of these muscles is also frequently performed, primarily due to their easy and direct accessibility, larger volume compared to other masticatory muscles, and greater contractile strength [4]. In such cases, intramuscular wet needling with local anesthetic injection is one of the most commonly used clinical procedures. This therapy is based on the hypothesis that local nerve blockade with anesthetics may be effective in treating MFP by inhibiting both muscle nociception and sympathetic nervous activity [8,9]. In these cases, local anesthetics without epinephrine are used to avoid local irritation of the muscle [10].

Considering the effect of nerve blockade, it has been suggested that anesthesia be applied directly to the nerves responsible for the sensory innervation of these muscles—specifically, the anesthetic blockade of the masseteric nerve and the deep temporal nerve. In these cases, it is recommended to perform the blockade using an anesthetic with epinephrine, since the infiltration is not directed toward the muscle but toward the nerve, thereby achieving a longer duration of anesthesia. Initially, these nerve blocks were performed separately, requiring two individual punctures [9,11]. However, in recent years, a new technique known as the “twin block” has been proposed, which allows anesthesia of both the masseteric and deep temporal nerves through a single extraoral puncture. This technique also effectively blocks the masseter and temporalis muscles, which are the primary contributors to masticatory MFP [10]. The twin block technique offers significant advantages over the traditional two-injection approach, as it achieves pain relief with a single puncture while also eliminating the risk of anesthetic leakage into the parotid fascia, which could inadvertently anesthetize the facial nerve [11].

While this novel technique, introduced by Quek et al. [11,12], is promising, its safe and effective application is critically dependent on a precise understanding of the complex regional anatomy. The foundational clinical papers by Quek et al. [11,13] and Ananthan et al. [10] describe the procedural protocol and its clinical efficacy, but a comprehensive review that synthesizes the essential anatomical information for this specific procedure is currently lacking. The specific novelty and contribution of this anatomical review, therefore, is to bridge that gap. This review aims to provide a consolidated and detailed anatomical guide by: (1) Describing the course and complex origins of the masseteric and deep temporal nerves (e.g., ADTN from the buccal nerve, PDTN from the temporomasseteric trunk) to provide the rationale for the single-puncture approach; and (2) Detailing their relationships with adjacent neurovascular structures to provide a clear “map” for procedural safety and risk avoidance. This synthesized knowledge is intended to enhance procedural accuracy, minimize complications, and facilitate the safe and effective application of the twin block technique.

## 2. Trigeminal Nerve

The trigeminal nerve (CN V), is the principal general somatic sensory nerve of the head, providing innervation to the face, teeth, oral cavity, nasal cavity, and the dura mater of the cranial cavity. It emerges from the lateral aspect of the pons in the mesencephalon, through a large sensory root and a smaller motor root [14,15].

The neurons of the sensory root form the trigeminal ganglion, from which the three main divisions of the trigeminal nerve arise: the ophthalmic nerve (V1), the maxillary nerve (V2), and the mandibular nerve (V3). The mandibular nerve is a mixed nerve (afferent and efferent), emerges from the middle cranial fossa through the foramen ovale and enters the infratemporal fossa. The motor root of CN V passes alongside the trigeminal ganglion without synapsing in it and joins the mandibular nerve. The motor fibers merge with the sensory fibers of the mandibular nerve as it passes through the foramen ovale to exit the skull [15].

The branches of the mandibular nerve are described in Table 1:

## 3. Course of the Masseteric Nerve

**Figure 1:**
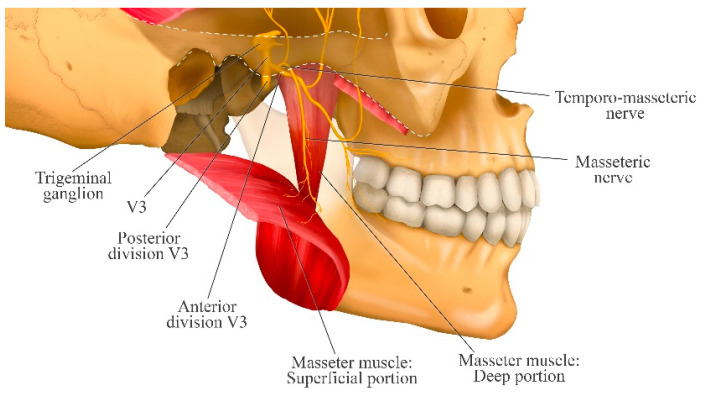
Course of the masseteric nerve and its branches in relation to the masseter muscle.

It arises from the anterior division of the mandibular nerve within the infratemporal fossa and gives rise to several branches, one of which is the temporomasseteric nerve. The temporomasseteric nerve then divides into two branches, giving rise to the masseteric and posterior deep temporal nerve; the masseteric nerve courses inferiorly and laterally, traverses the mandibular notch and then penetrates the deep surface of the masseter muscle [17]. Within the masseter muscle, the nerve divides into numerous branches that innervate both the superficial and deep portions of the muscle [18] (Figure 1).

Collar et al. [19] determined that the main branch of the masseteric nerve is located approximately 10 to 15 mm deep from the masseteric fascia and runs obliquely through the muscle for about 2 to 3 cm before branching. In this way, it provides both sensory and motor innervation to the masseter muscle.

Kaya et al. [20] dissected 12 hemifaces from 6 adult human cadavers and found that the nerve and its branches ran just above the periosteum in 8 cases, and between the superficial and deep portions of the masseter muscle in 4 cases. Between three and seven branches were observed, independently innervating the deep and superficial layers of the muscle.

Kim et al. [21] described four groups of nerve branches located between the superficial and deep portions of the muscle: posterosuperior, posteroinferior, anterosuperior, and anteroinferior. The anteroinferior group divides into four to five branches that innervate the majority of the superficial portion of the muscle. Among these, two or three branches pierce the superficial portion and reach its surface, specifically distributed over the inferomedial and anteroinferior regions. They identified the lower middle third of the masseter muscle as the area with the richest innervation by the masseteric nerve.

The origin of the nerve through the mandibular notch has been reported to lie 13.8 ± 4.5 mm inferior to the zygomatic arch, 10.6 ± 2.7 mm medial to the capsule of the temporomandibular joint (TMJ), and 7.8 ± 2.0 mm superior to the mandibular notch. Kaya et al. [20] further suggest that the masseteric nerve can generally be located approximately 1.0–1.5 cm below the zygomatic arch, 1 cm medial to the TMJ capsule, and 1 cm above the mandibular notch.

**Figure 2:**
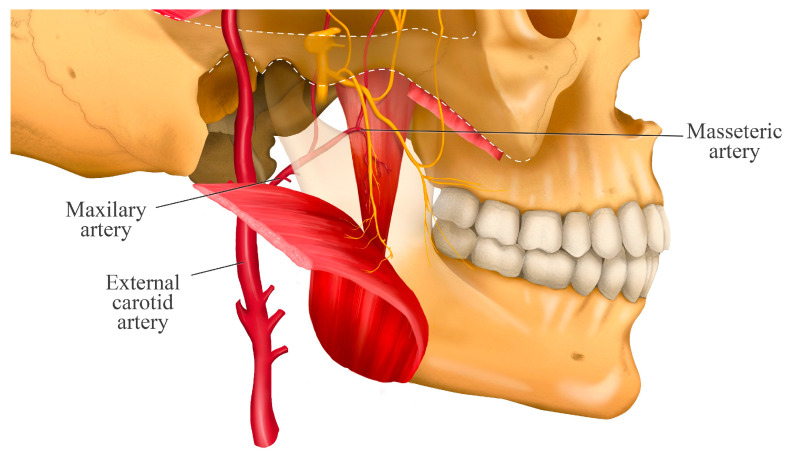
Relationship between the masseteric nerve and artery.

Additionally, Kaya et al. [20] observed that the branches of the masseteric nerve were accompanied by branches of the masseteric artery (Figure 2) and did not have a close relationship with other nerve branches.

Schematic representation of the course of the masseteric nerve from its origin in the anterior division of the V3, through its temporomasseteric branch, to its passage across the mandibular notch. The figure shows its entry into the deep surface of the masseter muscle, where it divides into multiple motor branches that innervate both the superficial and deep portions of the muscle. This arrangement explains the broad motor coverage and the nerve’s ability to modulate the activity of different segments of the masseter muscle.

Anatomical image illustrating the close relationship between the masseteric nerve and the masseteric artery along their course through the mandibular notch. Both structures run in close association toward the masseter muscle, highlighting the clinical importance of understanding this relationship when performing regional anesthesia techniques, as it helps reduce the risk of vascular complications.

## 4. Course of the Deep Temporal Nerves

**Figure 3:**
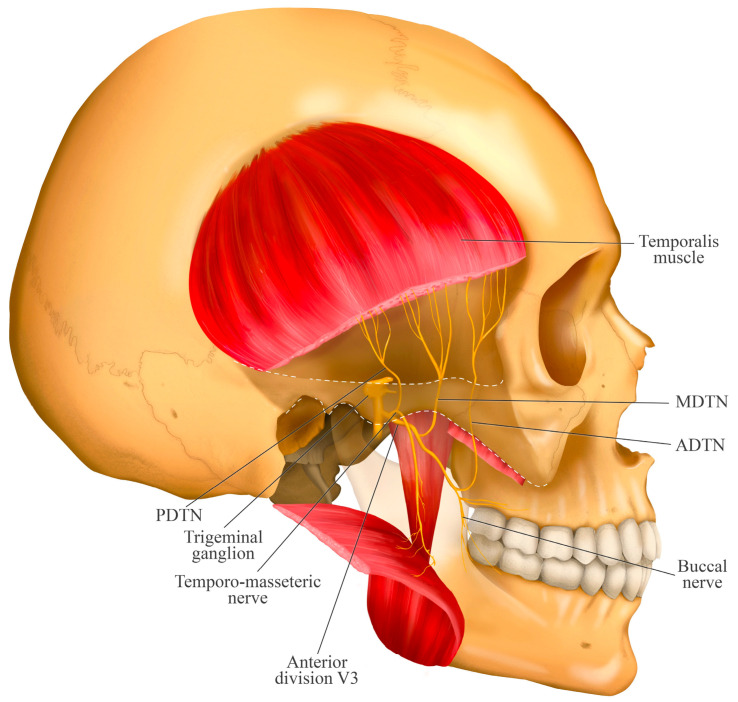
Origin and course of the deep temporal nerves.

The deep temporal nerves also originate from the anterior division of the mandibular nerve and pass between the roof of the infratemporal fossa and the superior surface of the upper head of the lateral pterygoid muscle. They divide into three nerves (Figure 3):

1. The anterior deep temporal nerve (ADTN) arises from the buccal nerve, which passes between the upper and lower heads of the lateral pterygoid muscle and distributes to the anterior portion of the temporalis muscle [22]. It has been reported that two to five branches may arise from this nerve [23].
2. The middle deep temporal nerve (MDTN) is a branch that is not always present [24]. When present, it branches off early from the main nerve (anterior division of the mandibular nerve). It typically travels along the superior surface of the lateral pterygoid muscle and often perforates its upper head to distribute into the middle portion of the temporalis muscle. The branching patterns are variable and have been classified into four types based on the number of branches: Type A (one branch), Type B (two branches), Type C (three branches), and Type D (four branches) [22].
3. The posterior deep temporal nerve (PDTN) arises independently from the temporomasseteric nerve. It travels along the superior surface of the upper head of the lateral pterygoid muscle and distributes to the posterior portion of the temporalis muscle [22]. It has also been described as having two to five branches [23].

This schematic illustrates the complex innervation of the temporalis muscle, showing the anterior (ADTN), middle (MDTN), and posterior (PDTN) deep temporal nerves. This anatomy is clinically critical, as the figure demonstrates the different origins of these nerves. Crucially, the temporo-masseteric nerve is the same trunk that gives rise to the masseteric nerve. This figure therefore highlights the close anatomical proximity between the nerves innervating the temporalis muscle and the masseteric nerve. Understanding this convergence of nerves within the infratemporal fossa is essential for accurate needle placement during processes such as regional anesthesia of the masticatory muscles.

## 5. Relationship with the Deep Temporal Artery

**Figure 4:**
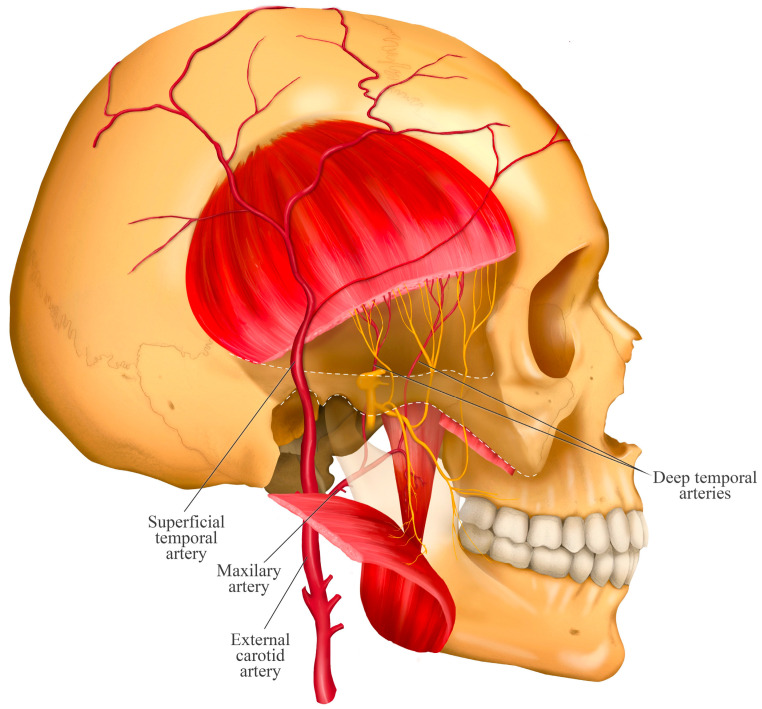
Relationship of the deep temporal arteries with the temporal nerves.

The temporalis muscle is supplied by the anterior deep temporal artery (ADTA), posterior deep temporal artery (PDTA), and middle deep temporal artery (MDTA) (Figure 4). The ADTA and PDTA are branches of the maxillary artery and accompany the deep temporal nerves through the infratemporal fossa. The MDTA, however, branches off from the superficial temporal artery at the level of the lower posterior margin of the zygomatic arch and enters the posterior portion of the temporalis muscle [12].

It has been reported that in most cases, after emerging from the infratemporal fossa, the PDTA lies deep and posterior relative to the posterior deep temporal nerve. In other cases, this artery may be located deep and anterior to that nerve [12].

Anatomical diagram illustrating the course of the anterior, and posterior deep temporal arteries and their close association with the homonymous nerves as they enter the temporalis muscle. The anterior and posterior deep temporal arteries are shown arising from the maxillary artery. This neurovascular proximity is key to understanding the potential risks of hemorrhage or anesthetic diffusion during invasive procedures in the region, as the deep temporal arteries represent the primary risk for hematoma during infiltration. This anatomical relationship dictates the need for careful aspiration before injection to prevent intravascular administration.

## 6. Isolated Masseteric Nerve Block

Protocol: The width of the mandibular ramus is assessed using the thumb and middle finger, referencing the anterior and posterior margins. The index finger is placed on the zygomatic arch and then moved downward to locate the mandibular notch. The puncture site is disinfected with 70% alcohol. The needle with the anesthetic is then inserted posterior to the index finger, forming an angle of approximately 40° in the coronal plane and 20° in the sagittal plane, aiming toward the neck of the mandibular condyle [8,9].

Dosage: 0.6–1.0 mL of local anesthetic, most commonly 2% lidocaine with 1:100,000 epinephrine to achieve a longer-lasting effect [6,9].

Advantages: The short-term therapeutic efficacy of masseteric nerve block for relieving MFP in the masseter muscle is promising. Authors suggest that this simple technique achieves both sensory and motor blockade of the muscle, potentially reducing muscle tone, producing analgesia to support desensitization processes, and silencing all the muscle’s trigger points [8].

Considerations: In rare cases, temporary loss of contraction of the orbicularis oculi muscle on the side of the block may occur due to diffusion of the anesthetic solution toward the facial nerve, causing temporary facial paralysis. Less common side effects include hematoma and/or infection [8].

## 7. Isolated Deep Temporal Nerve Block

Protocol: The anterior portion of the temporalis muscle is located by palpating just above the zygomatic bone, where a depression can be felt. The puncture site is disinfected with 70% alcohol. Deep to this portion of the temporalis muscle lies the greater wing of the sphenoid bone. The needle is directed toward this area until it contacts the sphenoid bone. An anesthetic is administered without removing the needle, as the deep temporal nerves run close to the bony surface [9].

Dosage: 0.6 mL of local anesthetic. As with the previous technique, the preferred anesthetic is 2% lidocaine with 1:100,000 epinephrine.

Advantages: This is a relatively simple and safe technique that produces both sensory and motor blockade of the muscle, potentially helping to reduce pain and sustained contraction in the temporalis muscle and its tendon. As a nerve block, it also silences all active trigger points [9].

Considerations: Hematoma, local swelling, and/or infection at the injection site may occur, though these are uncommon.

## 8. Twin Block Technique: Simultaneous Blockade of the Masseteric and Deep Temporal Nerves

The “twin block” technique is a novel regional nerve block involving a single extraoral injection that allows for the anesthesia of both the masseteric nerve and the anterior deep temporal nerve as they emerge from the infratemporal fossa [11]. This technique is proposed to affect both the sensory and motor components of the masseteric and anterior deep temporal nerves, resulting in pain reduction and improved mandibular function [10].

Protocol according to Quek et al. [13]: A 27- or 25-gauge (32 mm) carpule needle is used.

**Figure 5:**
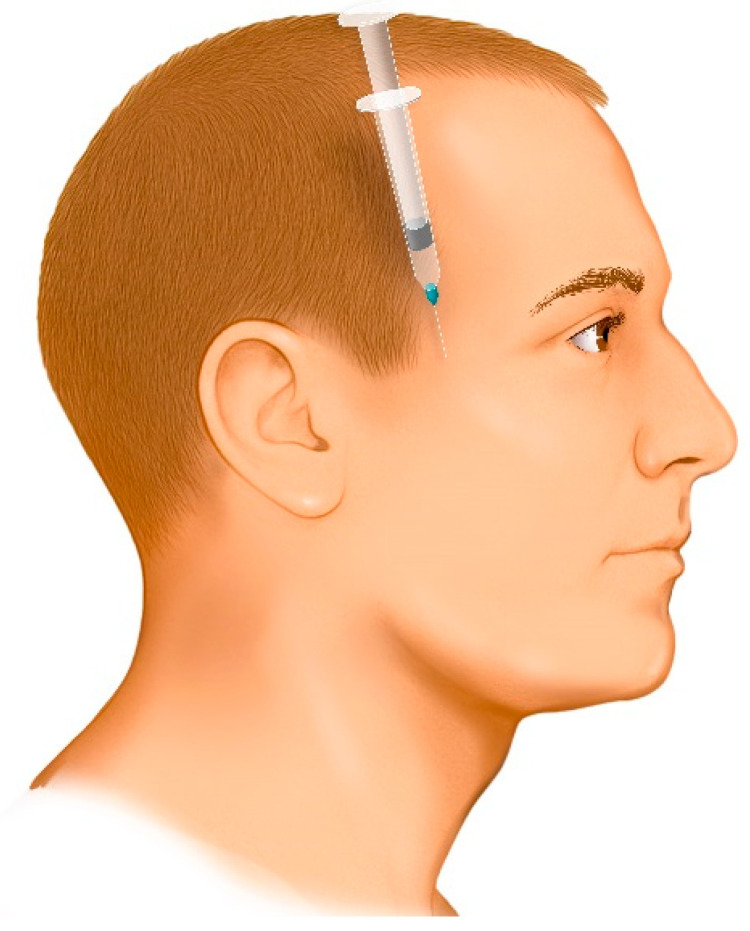
Injection site, direction and angle of the needle in the twin block technique.

1. The entry point is determined by palpating the superior margin of the zygomatic arch and locating the anatomical depression formed by the contact between the greater wing of the sphenoid bone and the superior border of the zygomatic process. This landmark is located approximately 10 mm posterior to the posterior border of the frontal process of the zygomatic bone (approximately the width of an index finger).
2. Disinfect the injection site using 70% alcohol.
3. The needle is directed downward between the zygomatic arch and the infratemporal fossa at an angle of 35° to 45° relative to the cranium (Figure 5). This angulation can be easily achieved by seating the patient in an upright position and aligning the needle to bisect the angle between the temporal bone (vertical plane) and the horizontal plane. If the needle is not angled properly, it may contact the coronoid process of the mandible, obstructing its advance. In such cases, the needle should be withdrawn slightly and redirected at a wider angle.
4. Advance the needle fully to its entire length.
5. Administer the anesthetic.
6. If a second injection is needed, a new sterile needle must be used to avoid cross-contamination.

This illustration highlights the critical importance of the entry landmark (the depression formed between the greater wing of the sphenoid bone and the superior border of the zygomatic process, approximately 10 mm posterior to the frontal process of the zygomatic bone) and the 35–45° downward angle. This specific angle is required to correctly access the space between the zygomatic arch and the infratemporal fossa and, crucially, to avoid contacting the coronoid process of the mandible, which could obstruct the needle’s advance.

Dosage: 1.7–1.8 mL of 2% lidocaine with 1:100,000 epinephrine [10,11].

Advantages: This is a straightforward technique that does not require the clinician to be an expert in identifying individual trigger points, as is necessary for trigger points injections (TrP) [10]. A single injection can provide both sensory and motor blockade of the masseter and temporalis muscles [11]. In cases with multiple trigger points in the masseter or temporalis, or both muscles, one injection can alleviate all pain [11]. It is also considered a safe technique, since vital structures within the infratemporal fossa remain beyond the reach of the needle due to the insertion angle and the protective position of the zygomatic arch, which prevents the horizontal trajectory required to reach deeper contents of the fossa.

**Figure 6:**
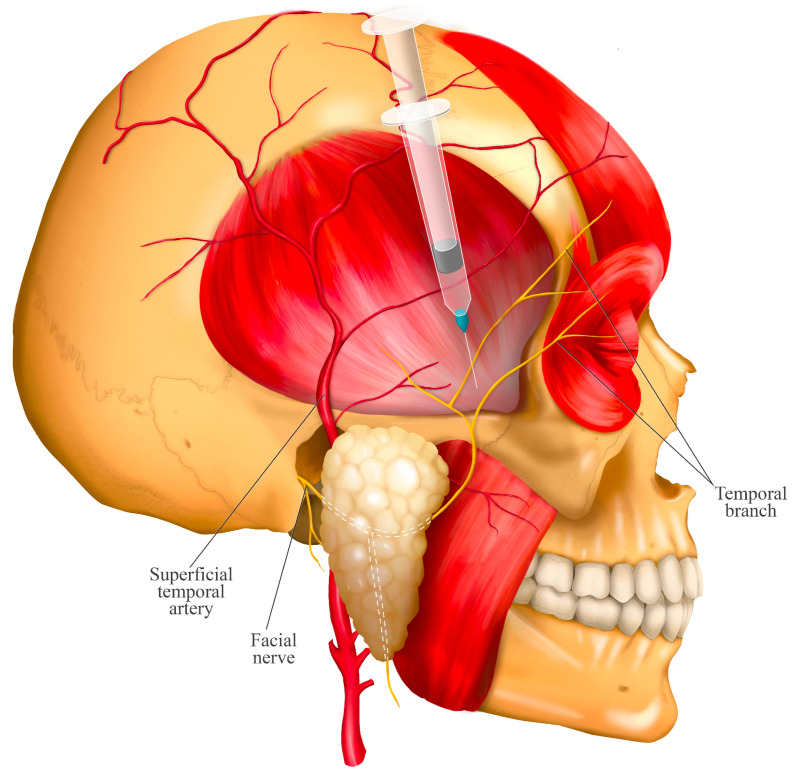
Relationship of the twin block injection site with neighboring nerve branches.

Considerations: There is a risk of inadvertently anesthetizing the facial nerve, potentially causing a temporary reduction in the blink reflex if the needle is inserted too superiorly, thereby affecting the temporal branches of the facial nerve (Figure 6). Similarly, a temporary facial paralysis may occur due to diffusion of the anesthetic solution, even if the needle is correctly placed but directed posteriorly. While these effects are not severe, it is important to inform the patient of this risk prior to the procedure [13]. As with any extraoral injection, there is a risk of infection, which underscores the importance of strict adherence to antiseptic protocol. Hematoma or damage to critical structures such as nerves or blood vessels is also possible; however, there are no major nerves or vessels near the injection site. The main vessels, such as the superficial temporal artery and the temporal branch of the facial nerve, are located superficially and posterosuperior to the injection site (Figure 6).

This schematic highlights the primary structures for risk avoidance. The posterosuperior and superficial location of the facial nerve (temporal branch) and superficial temporal artery dictates the safe injection zone, emphasizing why the needle must be inserted anterior to these structures and at the precise 35–45° angle to prevent temporary facial paralysis or vascular injury.

## 9. Discussion

Nerve blocks using local anesthetics have emerged as effective tools for the treatment of MFP, particularly due to their ability to intervene in nociception, sympathetic activity, and the motor component of the affected muscle [8]. In most cases where the masseter muscle is affected by MFP, the temporalis muscle is also involved. Therefore, blocking both nerves separately can be more invasive and carry greater risk, as it requires multiple injection sites, increasing patient discomfort and the likelihood of complications [6,10,11]. In this context, blocking the masseteric and deep temporal nerves has proven to be a useful strategy for managing masticatory pain, particularly in chronic MFP cases [4,10].

The twin block technique emerges as an anatomically and clinically advantageous alternative, enabling simultaneous blockade of the masseteric and deep temporal nerves through a single extraoral injection. Studies by Quek et al. [11,13] and Ananthan et al. [10] showed that this technique can offer faster, longer-lasting, and safer relief than conventional methods. It has demonstrated particular utility in chronic orofacial muscle pain, mandibular dislocations [9], and even as a diagnostic tool to distinguish between muscle, odontogenic, joint, or other origins of pain [25]. Compared to other interventional treatments (such as dry needling, wet needling, or TrP) the twin block technique is characterized by its truncal approach, acting globally on the sensory-motor innervation of the muscles [6,10]. MFP is a chronic muscular TMD involving both peripheral and central sensitization mechanisms, which result in heterotopic pain and autonomic activation phenomena. In this regard, nerve blocks help reduce pain perception and reverse plastic changes. Additionally, motor nerve blockade can decrease sustained contraction of taut bands [3] and simultaneously silence all trigger points in both muscles, achieving a broader therapeutic effect than isolated TrP [8,10].

This intervention has a direct impact on muscle tone reduction, peripheral and central pain desensitization, and the simultaneous silencing of multiple trigger points distributed across the masseter and temporalis muscles, including their tendons and fasciae [3,8,10]. The twin block technique has been described for the treatment of myogenic pain secondary to parafunctional habits [11], muscle pain following sustained mandibular opening, other causes of acute orofacial muscle pain, chronic orofacial muscular pain such as MFP, and various muscle-based TMDs. It has also been applied in cases of mandibular dislocation, helping to reduce pain and muscle contraction during manual reduction in the dislocated condyle [9].

Expanding on the clinical context, the recent study by Taşkesen & Cezairli [6] compared an isolated masseteric nerve block (MNB) against TrP injections with local anesthetic (LA) and dry needling (DN). Interestingly, their findings indicated that while all methods were effective, the MNB group showed a statistically smaller reduction in “pain on function” (PoF) scores compared to the LA group. On the other hand, the results for Maximum Mouth Opening (MMO) in the three groups showed a statistically significant improvement in MMO over time, with no significant difference between the groups. This suggests that the MNB was equally effective as LA and DN in restoring motor function. The authors themselves speculate on this mechanism, suggesting that the MNB, while perhaps less effective on immediate functional pain compared to LA and DN, is anticipated to reduce pain and increase the range of motion, thus taught bands dissolved in the long term, and the mouth opening increased [6]. These results must be interpreted with caution, as that study’s protocol focused only on the MNB, not the comprehensive “twin block”. As our review highlights, masticatory MFP frequently involves both the masseter and temporalis muscles. Therefore, the findings that an isolated MNB is less effective on pain are logical, as it leaves the temporalis muscle untreated. A case series by Kanti et al. [26] on 11 patients with chronic masticatory MFP demonstrated “expeditious and sustained efficacy” using the twin block, concluding it was a safe and effective approach. This provides initial support for the technique’s clinical utility. Furthermore, when compared to other common treatments, such as botulinum toxin (BoNT), the twin block offers distinct advantages. While BoNT has been shown to be an effective option for localized myofascial pain, offering significant pain reduction for up to 6 months, its onset is delayed (1–2 weeks), its cost is high, and it carries risks of excessive muscle atrophy [27]. The twin block, in contrast, offers immediate anesthetic feedback (useful for diagnosis) and can be repeated without these specific limitations.

Although reported adverse effects are generally mild and temporary (such as hematoma, paresthesia, or temporary facial paralysis). These can be minimized through proper injection technique and care. In the temporal region, the primary anatomical structure is the temporalis muscle. Needle insertion through this muscle is usually well tolerated by patients. Moreover, no major blood vessels or nerves are found near the needle’s trajectory [13]. The regional anatomy, as detailed in this study, supports the safety of the procedure. A thorough understanding of the course of the masseteric and deep temporal nerves, as well as their relationships with adjacent vascular structures, allows clinicians to perform the twin block technique with greater precision and safety. This knowledge is directly translational: understanding the posterosuperior location of the superficial temporal artery and facial nerve branches, for example, dictates the safe injection trajectory needed to avoid hematoma or temporary paralysis. The simplification of a potentially complex technique through clear anatomical landmarks is one of the primary strengths of this approach [13].

Despite the advantages of the anatomically guided technique, a significant limitation, and a critical area for future refinement, is the incorporation of modern imaging. The current twin block protocol relies on “blind” palpation of anatomical landmarks. While our review provides the anatomical “map” to make this approach safer, it still carries inherent risks, particularly the inadvertent puncture of the deep temporal or masseteric arteries. Recent studies, however, confirm that these nerve groups can be targeted with ultrasound (US) guidance. A clinical trial by Yildiz et al. [28] already demonstrates the clinical application of this concept, successfully using US guidance to perform pulsed radiofrequency (PRF) on the maxillary and mandibular nerves. The authors note that US-guided interventions are emerging as viable alternatives to traditional techniques and offer significant advantages. Specifically, US guidance is a “radiation-free alternative” and has the added benefit of visualizing structures, which is a key benefit in mitigating the exact risks of the ‘blind’ approach. Therefore, the development of a standardized US-guided protocol for the simultaneous twin block which targets these exact nerve groups is the clear next step. This would transition the twin block from a landmark-based technique to a safer, more precise, image-guided procedure, significantly strengthening its clinical applicability.

Lastly, future research should include controlled clinical trials with long-term follow-up to evaluate the duration of the therapeutic effect and its impact on patients’ quality of life. These trials would also be the ideal setting to compare the efficacy and safety profile of the anatomically guided twin block technique against other minimally invasive approaches, such as botulinum toxin injections or emerging ultrasound-guided nerve blocks.

## 10. Conclusions

The twin block technique, targeting the masseteric and deep temporal nerves, is an effective and minimally invasive alternative for managing masticatory myofascial pain, leveraging the anatomical proximity of these nerves for a single-puncture approach. This anatomical review provides the necessary “map” to enhance procedural accuracy and minimize complications, such as vascular injury or temporary facial paralysis.

While this anatomically guided technique is promising, and supported by initial case series, this review highlights two critical areas for future research, as detailed in the discussion. First, randomized clinical validation through large-scale controlled trials is necessary to confirm its long-term efficacy compared to other modalities, such as botulinum toxin or trigger point injections. Second, the development of standardized imaging-based guidance protocols, particularly using ultrasound, is essential to transition the ‘twin block’ from a landmark-based technique to a more precise, evidence-based procedure, further enhancing patient safety and efficacy.

## Figures and Tables

**Table 1:** Sensory and mixed branches of the mandibular nerve and their innervation.

Nerves	Innervation
Sensory branches of the mandibular nerve	
Auriculotemporal nerve	Skin of the temporal region, the ear, and part of the external acoustic pore of the skull, and part of the temporomandibular joint.
Lingual nerve	Anterior two-thirds of the tongue.
Inferior alveolar nerve	Lower gum and teeth, the terminal branch of which corresponds to the mental nerve that innervates the skin of the chin and lower lip.
Buccal nerve	Skin of the cheek and mucosa of the buccal region, and the gingiva and buccal groove of the lower molars and second premolar.
Mixed branches of the mandibular nerve (motor and sensory)	
Masseteric nerve *	Masseter muscle.
Deep temporal nerves (anterior, middle and posterior) **	Temporal muscle.
Pterygoid nerves (medial and lateral)	Medial and lateral pterygoid muscles.
Mylohyoid nerve	Mylohyoid muscle and anterior belly of the digastric muscle. This is a branch of the inferior alveolar nerve before it enters the mandibular canal.

Own elaboration based on what is described in Bathla & Hegde [14] and Schünke et al. [16]. * The detailed anatomical course is described in Section 3. ** The detailed anatomical course is described in Section 4.
